# Dynamical arrest for globular proteins with patchy attractions†

**DOI:** 10.1039/d4sm01275e

**Published:** 2025-01-08

**Authors:** Maxime J. Bergman, Tommy Garting, Cristiano De Michele, Peter Schurtenberger, Anna Stradner

**Affiliations:** a Division of Physical Chemistry, Department of Chemistry, Lund University PO Box 124 SE-221 00 Lund Sweden peter.schurtenberger@fkem1.lu.se; b Dipartimento di Scienze, Università degli Studi Roma Tre, Via della Vasca Navale 84 Roma Italy; c LINXS Institute of Advanced Neutron and X-ray Science, Scheelevägen 19 SE-223 70 Lund Sweden

## Abstract

Attempts to use colloid science concepts to better understand the dynamic properties of concentrated or crowded protein solutions are challenging due to the fact that globular proteins generally have heterogeneous surfaces that result in anisotropic or patchy contributions to their interaction potential. This is particularly difficult when targeting non-equilibrium transitions such as glass and gel formation in concentrated protein solutions. Here we report a systematic study of the reduced zero shear viscosity *η*_r_ of the globular protein *γ*_B_-crystallin, an eye lens protein that plays a vital role in vision-related phenomena such as cataract formation or presbyopia, and compare the results to the existing structural and dynamic data. Combining two different tracer particle-based microrheology methods allows us to precisely locate the line of kinetic arrest within the phase diagram and characterize the functional form of the concentration and temperature dependence of *η*_r_. We show that while our results qualitatively confirm the existing view that this protein can be reasonably well described using a coarse-grained picture of a patchy colloid with short range attractions, there are a number of novel findings that cannot easily be understood with the existing simple colloid models. We demonstrate in particular the complete failure of an extended law of corresponding states for a description of the temperature dependence of the arrest line, and discuss the role that transient clusters play in this context.

## Abstract 2

Dynamic properties of γ_B_-crystallin are incompatible with a colloid model for attractive ellipsoids that reproduces static data and phase diagrams.

## Graphical abstract

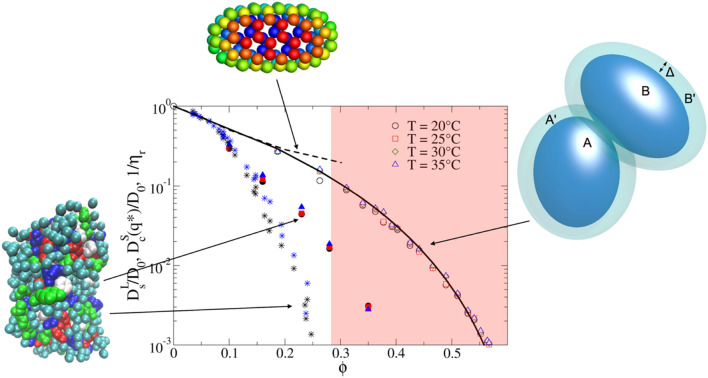

## 1. Introduction

Protein phase behavior and the existence of liquid–liquid phase separation in protein solutions and mixtures have entered again into the focus of the life sciences and biophysics community.^1^ For example, Klosin *et al.* have investigated how the cell ensures minimal effect from protein concentration fluctuations, caused by the stochasticity of protein expression.^2^ They hypothesized that protein concentrations in the cytosol are kept constant *via* compartmentalization of excess protein, driven by phase separation. These authors were indeed able to demonstrate that liquid droplets, formed through phase separation, can effectively reduce expression noise for their engineered protein system. Evidently, equilibrium phenomena such as liquid–liquid phase separation can underlie intra-cellular control mechanisms.^3^ However, non-equilibrium processes such as dynamical arrest could threaten protein regulation: if an arrest occurs during phase separation, the resultant phases will contain significantly different protein concentrations than anticipated.^4^

Recent evidence that phase transitions in living cells may represent an important mechanism in cellular organization has spurred renewed interest in exploring analogies to colloid solutions. Liquid–liquid phase separation has been studied extensively in the past, often driven by its connection to the occurrence of so-called protein condensation diseases.^5,6^ Initially, protein phase behavior was found to have clear analogies to colloids interacting with short range attractions. A key observation was for example that the location of the binodal and spinodal in protein solutions was also following the so-called extended law of corresponding states (ELCS).^7,8^ The ELCS provides a convenient link between the reduced second virial coefficient 
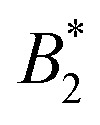
 as a measure of the interaction strength obtained at low concentrations and the predicted phase separation at high concentrations.^9^ In combination with isotropic potentials such as for example a combination of excluded volume repulsion and short range attractions, and applying a non-equilibrium self-consistent generalized Langevin equation theory, this approach has been able to reproduce the experimentally observed interplay between liquid–liquid phase separation and dynamical arrest in protein solutions quite successfully.^10^ However, despite the considerable insight provided by the ELCS combined with isotropic colloid models, this theoretical framework was subsequently found to be limited in application: proteins often have heterogeneous surfaces leading to anisotropic and patchy interactions, and recent efforts have revealed that patchy colloid models harbour more predictive power when it comes to static properties of protein solutions.^11–14^ Moreover, it was also found that more refined interaction models that include attractive patches and/or shape anisotropy are particularly important for a correct description of the dynamic properties of protein solutions.^3^ Unfortunately, there exists only limited information on the effects of patchy interactions on dynamic properties and the related non-equilibrium liquid–solid transitions. Furthermore, achieving a structure-based assignment of the location of patches and their interaction potential remains difficult, and requires for example a more refined coarse-graining strategy that starts from a molecular description of the protein.^15,16^

Attractive patches appear to be responsible for an arrest transition at much lower protein concentrations than typically found for classical colloids interacting primarily through centrosymmetric interaction potentials, and arrest seems to be linked to the formation of transient clusters or networks.^17–20^ Progress is currently limited by the lack of information available on the influence of patchy attractions on macroscopic flow properties in solutions of globular proteins such as the zero shear viscosity. This is not only crucial in our strive for understanding dynamic processes in the cellular environment, but also important in current attempts to develop high concentration biological formulations for drug delivery, amongst others. Patchy protein–protein interactions can lead to a dramatic increase of the viscosity of such formulations even at moderate volume fractions due to the formation of transient clusters, thus strongly impinging syringeability.^15^

Here we focus on the lens protein *γ*_B_-crystallin, which is known to exhibit liquid–liquid phase separation, with a critical concentration around *c*_crit_ ≈ 220–240 mg mL^−1^, or a critical volume fraction of *ϕ*_crit_ ≈ 0.15–0.17.^17,21,22^*γ*_B_-crystallin is known to play a key role for example in cataract formation and presbyopia.^5,18,23–25^ This connection between liquid–liquid phase separation in solutions of eye lens proteins and the formation of cataracts has led to a systematic study of the phase diagram of this protein.^11,17,21,22,26–28^ The application of coarse-grained colloid models such as hard spheres with short range attractions, patchy colloids or hard ellipsoids with short range attractions resulted in fairly good agreement with the experimentally determined static properties of *γ*_B_-crystallin solutions.^11,17,26^ However, subsequent studies also revealed that the dynamic properties of *γ*_B_-crystallin solutions are much more complex and cannot be reproduced with simple colloid models based on isotropic interaction potentials.^18^

The dynamic properties of *γ*_B_-crystallin solutions have so far mainly been investigated by dynamic light scattering (DLS) and neutron spin echo spectroscopy (NSE), both characterizing collective diffusion at different characteristic time and length scales.^17,18,29^ DLS looks at collective diffusion on length scales of a few hundred nanometers, which for concentrations around the critical concentration is dominated completely by critical slowing down, resulting in a strong temperature and concentration dependence.^17,29^ At higher concentrations, DLS reveals the presence of a large noncritical background contribution that results in an unusual logarithmic rather than exponential decay of the intermediate scattering function (ISF), presumably due to the combined effects of critical slowing down and an approaching arrest transition.^17^ NSE probes dynamics at nanometre length scales and the results show a dramatic reduction in collective cage diffusion around the nearest neighbor distance for increasing concentration. This observation, combined with the estimate of the location of the arrest line based on the indication of non-ergodicity in DLS, suggests that the dynamical arrest is driven by the formation of transient clusters and networks created by attractive patches rather than an isotropic potential.^17,18^ Unfortunately, we currently lack information on the concentration and temperature dependence of the solution viscosity as a direct measure of the macroscopic flow properties of *γ*_B_-crystallin solutions.

Therefore we now extend the existing dynamical information by probing the zero shear viscosity of *γ*_B_-crystallin for several temperatures using two passive microrheology techniques, combined with a new approach for preparing highly concentrated and solid-like samples.^30^ These results are compared to repulsive and attractive colloid models, where we find that the concentration dependence of the reduced viscosity *η*_r_ = *η*_0_/*η*_s_, where *η*_0_ is the zero shear viscosity of the protein solution and *η*_s_ that of the solvent, follows a power law characteristic of isotropically attractive spheres – although a clear signature of the attractive patches can be observed in the early onset of the arrest transition. Surprisingly, the observed concentration dependence of *η*_r_ appears independent of temperature. The arrest line is thus also temperature-independent above the binodal, even though interactions between *γ*_B_-crystallins have already been shown to change significantly in this temperature range.^8^ Moreover, a weak contribution to *η*_r_ close to the critical point arises from critical behavior, which does not appear to follow the laws of universality, hinting at additional contributions to the viscosity. We finally formulate a scenario harmonizing our current findings with previous conclusions and discuss a possible generalization of these results to other proteins interacting *via* patchy interactions. Our findings are important not only to better understand the cellular environment, but also to improve the formulation of high concentration biopharmaceuticals.

## 2. Results and discussion

**Fig. 1:**
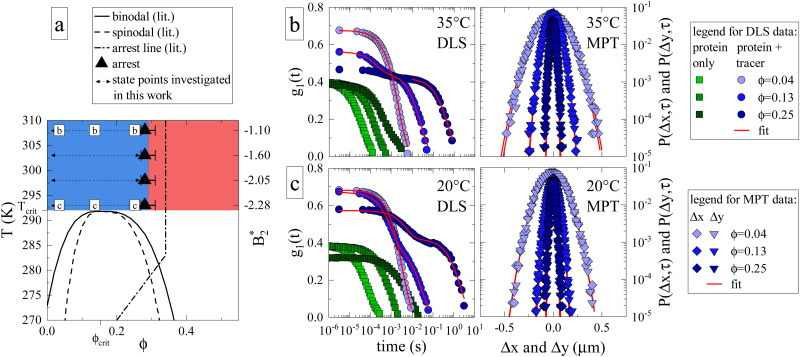
(a) Adaptation of the state diagram for *γ*_B_^17^ with added results from the current work. This previous work determined the binodal (solid line), spinodal (dashed line) and arrest line (dashed dotted line). We now add the range of state points investigated in this work (arrows) and the position of the arrest transition based on the MCT-like power law (triangles) and evaporation experiments (horizontal bars). Also indicated are the state points shown in panels (b) and (c). The measured values of the reduced second virial coefficient 
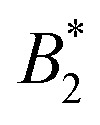
 are shown on the right figure axis at the corresponding temperatures (from ref. 8). (b) Representative experimental data of three samples at 35 °C for DLS (left) and MPT (right) measurements. The DLS panel displays the field correlation function *g*_1_(*t*) for protein samples with (blue circles) or without (green squares) embedded tracer particles. Red lines represent the fit to the protein + tracer mixtures. A decimated number of data points is shown for clarity, with the full curve shown in Fig. S1 (ESI†). The MPT panel depicts the Van Hove function, which indicates the range of movement for the tracer particles in both *x*- and *y*-direction (diamonds and downwards triangles), with corresponding Gaussian fits (red lines). (c) Same type of data as shown in panel (b) but for samples measured at 20 °C, close to *T*_crit_.

In order to gauge the macroscopic flow properties of *γ*_B_-crystallin, a large number of protein solutions were prepared, covering a broad range of volume fractions (0.035 ≤ *ϕ* ≤ 0.25) and temperatures (20 °C ≤ *T* ≤ 35 °C), where we profited from a novel evaporation technique to achieve protein volume fractions beyond the arrest transition. Tracer particles were added to *γ*_B_-crystallin samples, and their motion was recorded using DLS-based and MPT-based (multiple particle tracking) microrheology. The latter technique relies on tracking the individual fluorescent tracer particles using videos created with confocal laser scanning microscopy (CLSM), whereas the former rests on the light scattering caused by tracers (see Materials and Methods section for further information). The concentration and temperature range were chosen based on the previously published experimental state diagram (Fig. 1a, adapted from ref. 17), in order to cover conditions where the samples are in the previously located fluid phase above the critical temperature *T*_crit_, and extending to the estimated location of the arrest line. Within the chosen temperature range, earlier static light scattering (SLS) and DLS measurements have demonstrated a dramatic variation of the isothermal compressibility and the dynamic correlation length describing the decay of long wavelength concentration fluctuations by almost two orders of magnitude, in particular close to the critical isochore at *ϕ*_crit_.^17^

We first determine how the proximity to the critical point (*ϕ*_crit_ = 0.154, *T*_crit_ ≈ 19 °C)^17^ and the related critical opalescence and critical slowing down experienced by the protein solution affects the microrheological methodology. For this reason, Fig. 1b displays typical results from microrheology measurements taken at 35 °C, *i.e.* far away from *T*_crit_, and Fig. 1c showcases data at 20 °C. Each panel includes three state points: *ϕ* = 0.04 < *ϕ*_crit_, *ϕ* = 0.13 ≈ *ϕ*_crit_ and *ϕ* = 0.25 > *ϕ*_crit_. In the DLS-based approach, the field autocorrelation function *g*_1_(*t*) is obtained from the measured intensity autocorrelation function *g*_2_(*t*), which depends on the scattering emanating from both the protein and the tracers. In MPT, the probability of a tracer displacement is expressed using the Van Hove function. Both microrheology approaches clearly display a retardation of tracer motion (blue symbols in Fig. 1b and c) with increasing protein volume fraction, indicated in DLS as a shift to longer decay times while in MPT as a narrowing of the displacement distribution.

Whereas the MPT method provides consistent results, regardless of the location of the state point, this is different for DLS-based microrheology. Here the increased scattering due to protein critical behavior clearly complicates the analysis of the DLS tracer data at any given temperature. In the diluted regime, there is no significant contribution from protein scattering. The decay of *g*_1_(*t*) is dominated by the tracer signal, resulting in a single, easily analysed decay (light blue circles, Fig. 1b and c). However, close to *ϕ*_crit_ critical scattering increases the protein signal, and a critical slowing down moves the corresponding decay from the protein solution to much larger decay times. This is illustrated by DLS measurements on pure protein samples (green squares, Fig. 1b and c). The effect on *g*_1_(*t*) as measured on protein plus tracer mixtures (medium blue circles, Fig. 1b and c) is therefore not surprising: the overall *g*_1_(*t*) exhibits two decay processes, which nearly merge. The first is associated with the temporal decay of critical protein concentration fluctuations and the second originates from the tracer diffusion. We attempt to enhance the tracer signal by using sufficiently high tracer particle concentrations to effectively drown out the intrinsic protein scattering contribution to *g*_1_(*t*), with any possible multiple scattering suppressed by the use of a so-called 3D DLS technique.^31^ Regardless, the overlap between the two scattering contributions mitigates accurate analysis of the tracer decay and the thus obtained values of *η*_r_ carry a larger uncertainty.

Protein contributions to scattering and *g*_1_(*t*) remain significant beyond *ϕ*_crit_. At high temperatures, the two parts of the decay are sufficiently separated to analyse the tracer motion only (Fig. 1b, dark blue circles). At temperatures close to *T*_crit_, the interplay between critical slowing down and approaching dynamical arrest leads to the previously mentioned unusual logarithmic decay of *g*_1_(*t*),^17^ which is not easily analyzed (Fig. 1c, dark blue circles). Evidently, DLS measurements close to either *ϕ*_crit_ or *T*_crit_ are impacted tremendously by (general) critical protein behavior. In contrast, MPT-based microrheology relies on fluorescence, and is not at all affected by the increased scattering contribution of the proteins. Using DLS-based microrheology to explore the flow properties of protein solutions in the vicinity of a critical point could thus lead to an overestimation of *η*_r_ and suggest a large, additional critical contribution to the zero shear viscosity, which is however not apparent in the MPT data (see also Fig. S1 and S2 for further illustration, ESI†).

**Fig. 2:**
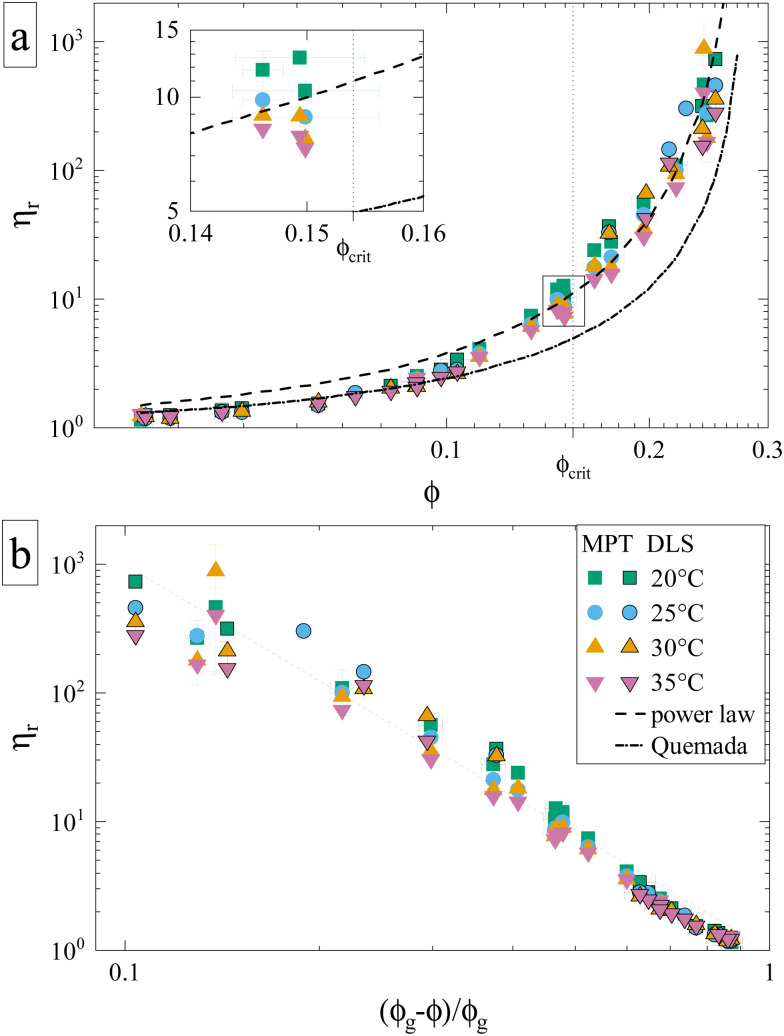
Reduced sample viscosities at four different temperatures obtained using DLS (framed symbols) and MPT (unframed symbols), ranging from just above the critical temperature (20 °C) to 35 °C. The lines indicate fits, either to the Quemada expression for hard spheres (dashed-dotted) or a power law used for attractive spheres (dashed) with an exponent *γ* = 3.1 and *ϕ*_g_ = 0.28 (see text). Both panels contain the same data represented in a different way, and the legend applies to both. (a) *η*_r_ plotted *versus* volume fraction *ϕ*. Inset: Close-up of datapoints at *ϕ*_crit_, which are (partially) shown in Fig. 4. (b) *η*_r_ plotted *versus* the reduced volume fraction *ε* = (*ϕ*_g_ − *ϕ*)/*ϕ*_g_, which allows for a better comparison to the power law.

Having established the appropriate methodology for measuring *η*_r_ for *γ*_B_-crystallin solutions over the required concentration and temperature range, we next present the results from the four concentration series at all temperatures 20 ≤ *T* ≤ 35 °C (Fig. 2). All *η*_r_ values superimpose on a single curve within the experimental uncertainty, and no effect of temperature on the functional dependence can be seen, indicating a temperature-independent approach to and location of the arrest transition around *ϕ*_g_ ≈ 0.28. Good correspondence is found between the data points from the two techniques when omitting the DLS-based data around the critical concentration, confirming our previous conclusions on the broad applicability of such microliter-based techniques for protein characterization.^30^

Such an absence of temperature dependence for the concentration dependence of *η*_r_ and for the location of the arrest line *ϕ*_g_ of *γ*_B_-crystallin is quite unexpected. Previous work revealed that *γ*_B_-crystallin is an attractive system with a pronounced *T*-dependence of the reduced second virial coefficient 
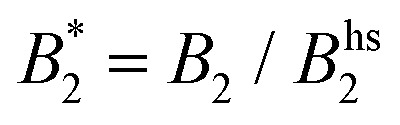
, where *B*_2_ is the second virial coefficient of the protein and *B*^hs^_2_ that of a reference hard sphere system.^8^ The ELCS asserts a connection between 
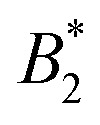
 and the location of the binodal and spinodal.^9^ Recently, it has successfully been extended to include the dependence of non-equilibrium phenomena, such as an arrest line, on 
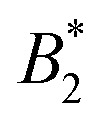
.^32^ Indeed, comparable work for synthetic colloids with short range attractions have reported a change of the arrest transition from 0.18 ≤ *ϕ*_g_ ≤ 0.36 upon a change of temperature or 
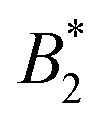
 by 
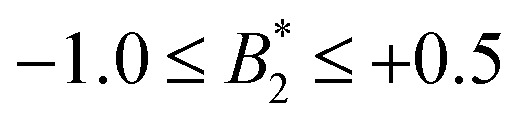
.^32^ In addition, a recent simulation study showcases that the ELCS also applies to patchy particles: the binodals of particles with the same patch distribution, but different types of patches, collapse when plotted as a function of 
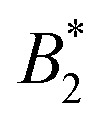
.^33,34^ In our case, 
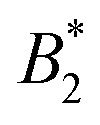
 increases from 
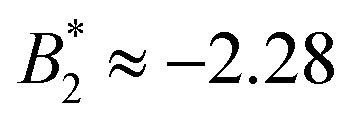
 at 20 °C to 
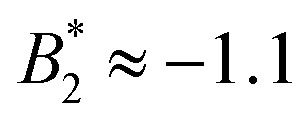
 at 35 °C,^8^ but against expectations we observe no measurable change in *ϕ*_g_ (see Fig. 1). This *T*-independence of *ϕ*_g_ is reminiscent of effective hard spheres rather than attractive particles, but of course the observed values of *ϕ*_g_ are much too low for an effective hard sphere behavior.

An attempt to fit the concentration dependence of the reduced viscosity with classical hard sphere models such as Quemada,^35^ Krieger–Dougherty,^36^ Mooney^37^ as well as a modified Vogel–Fulcher–Tammann (VFT)^38,39^ description also clearly fails. Such (empirical) models have been used with varying success, especially in combination with the introduction of an effective size to approximate the protein as a quasi-spherical colloid. Yet, the viscosity behavior of *γ*_B_-crystallin is clearly affected by attractions, as none of the aforementioned models yielded a satisfying agreement, with Quemada shown as an example (Fig. 2a, dashed-dotted line).

Theoretical work on hard sphere and attractive systems using mode coupling theory (MCT) and computer simulations predicts a power-law dependency of the reduced viscosity *η*_r_ ∼ *ε*^−*γ*^ in the vicinity of the arrest transition, where *ε* is a reduced order parameter defined as *ε* = (*X*_g_ − *X*)/*X*_g_, with *X* given either by the volume fraction *ϕ* or the strength of the attraction.^40^ The value of *γ* then depends on the interaction between particles, with *γ* = 2.8 for hard spheres, and values *γ* ≥ 3 for attractive particles.^40,41^ The viscosity data obtained for *γ*_B_-crystallin solutions is indeed well reproduced at higher concentrations with a power law exponent *γ* = 3.1 ± 0.1 when using *ϕ*_g_ = 0.28 (see Fig. 2a, dashed line). The range over which this power law appears to be valid is surprisingly large. While for the hard sphere-like eye lens protein *α*-crystallin an MCT-like power law with an exponent of *γ* = 2.8 was found to reproduce the data only for *ε* ≤ 0.13,^42^ here the data follows a power law over a much more extended range *ε* ≤ 0.6 (Fig. 2b). Similar differences were also found in the simulation study, with a validity of the power law for *ε* ≤ 0.07 for hard spheres and *ε* ≤ 0.34 for attractive particles.^40^

**Fig. 3:**
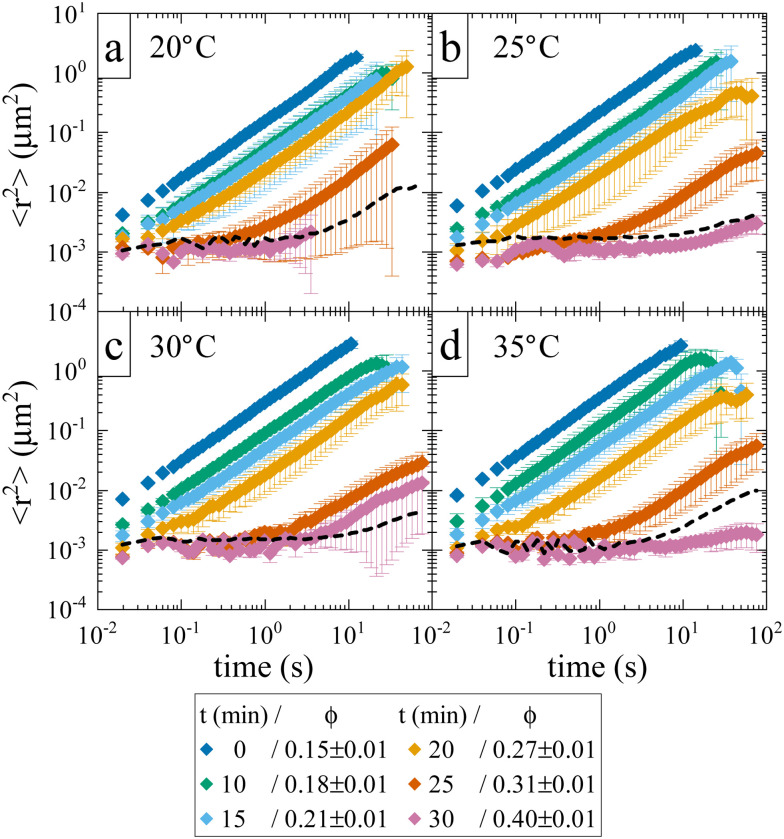
Average tracer MSDs extracted from MPT-microrheology on solid-like samples, achieved *via* evaporation. Each panel shows data obtained at a different temperature: (a) 20 °C; (b) 25 °C; (c) 30 °C; (d) 35 °C. The legend indicates the evaporation time and the volume fraction of the samples. The dashed line indicates a reference measurement of tracer particles fixated in a polymer matrix and displays instrumental drift.

While the location of the arrest line has so far only relied on an analysis of the concentration dependence of the reduced viscosity, here we now confirm the existence of an arrested solid-like state at volume fractions *ϕ* > *ϕ*_g_ using evaporation experiments. This allows us to directly probe volume fractions beyond *ϕ*_g_ with MPT-based microrheology that are not accessible using standard sample preparation procedures for dense protein solutions. In an earlier work,^30^ we demonstrated the efficiency of such an experiment to obtain otherwise inaccessible volume fractions. Although the evaporation procedure results in a somewhat heterogeneous concentration profile within the sample cell, this can be taken care of by ensuring that confocal measurements are performed at several different locations within the sample. Fig. 3 shows the average MSD of tracers as a function of evaporation period or average volume fraction *ϕ*. We see the expected slowing down of the tracer diffusion with increasing *ϕ* and the formation of a completely arrested sample at around *ϕ*_g_ ≈ 0.27–0.31. In the arrested samples any apparent residual motion of the tracer particles is due to macroscopic collective drift (Fig. S3, ESI†), primarily caused by the instrument (black dashed line in Fig. 3). The resulting location of the arrest transition is indicated in Fig. 1 by horizontal bars and is in good agreement with the value of *ϕ*_g_ ≈ 0.28 obtained from the power law fit to the viscosity data, and at slightly lower volume fractions than what was previously reported in the literature based on DLS measurements.^17^

**Fig. 4:**
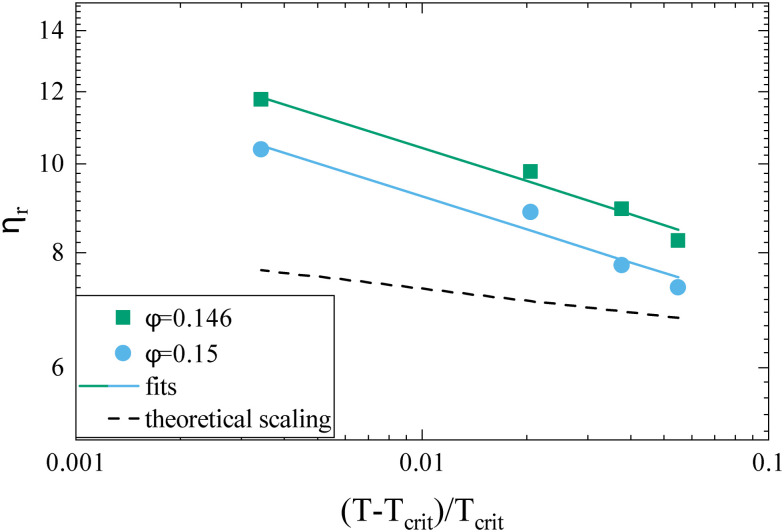
Reduced viscosity *η*_r_*versus* reduced temperature *ε*_*T*_ = (*T* − *T*_crit_)/*T*_crit_ for two concentrations *ϕ* = 0.146 (green squares) and *ϕ* = 0.150 (blue circles), respectively. Also shown are the fitted power laws *η*_r_ ∼ *ε*_*T*_^−*x*^ (solid lines) and the theoretical slope for the scaling relationship for binary critical fluids with *x* = 0.043 (dashed line).

While the overall functional form of the concentration dependence of *η*_r_ appears independent of temperature, a closer look at the MPT-based microrheology nevertheless indicates a systematic weak temperature dependence at concentrations close to the critical concentration *ϕ*_crit_ (Fig. 2a, inset and Fig. S4 in the ESI†). In phase-separating systems, *η*_r_ encompasses a non-critical component *η*_noncrit_ as well as a critical component *η*_crit_. The power law-like approach to the arrest, as discussed previously, describes *η*_noncrit_. Any observed divergence from this power law at concentrations close to *ϕ*_crit_ should thus correspond to the critical contribution *η*_crit_. Universal scaling laws for binary fluids dictate that upon approaching the critical point, *η*_crit_ scales as *η*_crit_ ∼ *ξ*^−*x*_*η*_^, where *ξ* is the correlation length, related to the reduced temperature *ε*_*T*_ = (*T* − *T*_crit_)/*T*_crit_ by a similar scaling relation *ξ* ∼ *ε*_*T*_^−*ν*^.^43,44^ Using literature values *x*_*η*_ = 0.068 and *ν* = 0.63, this results in an asymptotic scaling law of *η*_crit_ ∼ *ε*_*T*_^−0.043^. Rather than the theoretical exponent 0.043, we instead find experimentally a value of 0.12 ± 0.02 for volume fractions in the vicinity of *ϕ*_crit_ (Fig. 4), suggesting another, unknown contribution to the zero shear viscosity. Clearly, the possible existence of additional contributors to the zero shear viscosity in the vicinity of a critical point invites further research. Following the viscosity along a critical isochore requires measurements over a large range of temperatures with high accuracy and resolution. The temperature control in our MPT microrheology experiment is unfortunately not good enough for such a study, so there is obviously a need for future experiments. Nevertheless, our results indicate a possibly stronger temperature effect than what one would expect from a comparison with simple fluids.

We are thus confronted with a number of findings that partly seem contradictory. On the one hand, typical for attractive and patchy systems, *γ*_B_-crystallin solutions show an arrest transition at a low volume fraction *ϕ*_g_ ≈ 0.28. Starting as low as *ϕ* = 0.13, the concentration dependence of *η*_r_ can be described with an MCT-like power law with an exponent *γ* = 3.1 ± 0.1 that is slightly higher than the value for hard spheres (2.8), in agreement with simulation data for weakly attractive colloids.^40,41^ On the other hand, we find no measurable temperature dependence of the location of the arrest transition *ϕ*_g_, despite the fact that the reduced second virial coefficient 
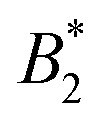
 varies considerably across the temperature range.^8^ This indicates an effective hard sphere- or hard ellipsoid-like behavior, despite the fact that all existing structural and dynamic properties are in agreement with a short range attractive and patchy potential.^11,17,18^ This is also in line with the earlier findings for proteins undergoing an arrested spinodal decomposition.^4,10^ These studies demonstrated that the binodal and spinodal for liquid–liquid phase separation would follow an extended law of corresponding states, *i.e.*
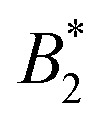
 could be used as an effective temperature to rescale the experimental phase boundaries obtained for different solution conditions. However, the location of the arrest line within the unstable region of the phase diagram was determined rather by the contact value of the potential and would not rescale with 
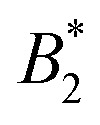
.^4,10^

At present we can only speculate about this peculiar arrest behavior. We currently believe that the arrest transition is caused by transient clusters formed by the attractive patches. These transient (and polydisperse) clusters are characterized by an open structure with low internal density. While the patch attraction leads to the formation of such clusters, the clusters themselves behave as effective hard spheres, driving the observed glass transition at *ϕ*_g_ ≈ 0.28. In a recent simulation study, the existence of such transient clusters was proposed in order to explain the cage diffusion behavior of *γ*_B_-crystallin.^18^ Cage diffusion is represented by the short time collective diffusion coefficient *D*_s_(q*) measured at a *q*-value q*, corresponding to the nearest neighbor peak position in *S*(*q*). Measured over a comparable temperature range as used in this study, Bucciarelli *et al.* found that cage diffusion was not affected by temperature, but rather exhibited a dramatic slowing down upon increased *γ*_B_-crystallin concentration. This concentration-induced impact on cage diffusion was shown to be best reproduced with a patchy attractive model introducing transient clusters.^18^ A similar model has recently been shown to reproduce the strong concentration dependence in concentrated solutions of self-assembling monoclonal antibodies, where opposite charges on different parts of the antibody would create attractive patches as a driving force for self-assembly.^15^

**Fig. 5:**
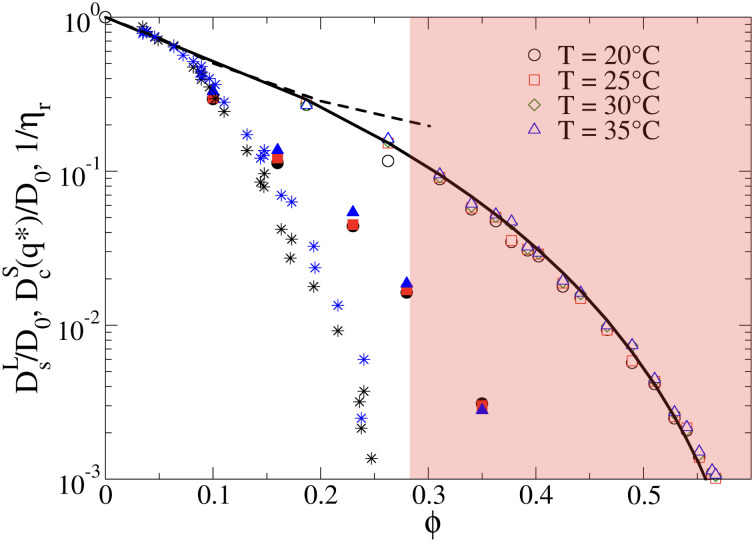
The inverse of the reduced viscosity 1/*η*_r_*versus* volume fraction *ϕ* for two temperatures *T* = 20 °C (black stars) and *T* = 35 °C (blue stars), respectively, and the concentration dependence of the normalised short-time cage diffusion coefficient *D*^S^_c_(q*)/*D*_0_ (data taken from ref. 17) for three temperatures *T* = 20 °C (black filled circles), *T* = 25 °C (red filled squares) and *T* = 35 °C (blue filled triangles). Also shown are the results from computer simulations of weakly attractive hard ellipsoids with dimensions mimicking *γ*_B_-crystallin. The data for the normalized long-time self diffusion coefficient *D*^L^_s_/*D*_0_ are given by the open symbols for 4 different temperatures (*T* = 20 °C (open black circles), *T* = 25 °C (open red squares), *T* = 30 °C (open green diamonds), *T* = 35 °C (open blue triangles)) and are well-reproduced by a power law of the form *D*^L^_s_/*D*_0_ = [(*ϕ*_g_ − *ϕ*)/*ϕ*_g_]^*γ*^ (solid line), with *ϕ*_g_ = 0.66 and *γ* = 3.7. Also shown are the results from simulations for weakly attractive ellipsoids including hydrodynamic interactions for *D*^S^_c_(q*)/*D*_0_ taken from ref. 45 given by the black dashed line. The red shaded region indicates macroscopic arrest.

The importance of patchy attractions in determining short- and long-time dynamic properties is further illustrated in Fig. 5. Here we compare experimental results for the concentration dependence of the inverse of the relative viscosity 1/*η*_*r*_ and the normalised short-time cage diffusion coefficient *D*^S^_c_(q*)/*D*_0_ with the predictions for the reduced long-time self diffusion coefficient *D*^L^_s_/*D*_0_ from computer simulations based on a model of attractive ellipsoids. This colloid model had previously been shown to quantitatively reproduce static properties such as the temperature and concentration dependence of the osmotic compressibility or the location of the binodal for liquid–liquid phase separation.^17,45^

The simulations and the underlying model of weakly attractive ellipsoids with dimensions closely resembling *γ*_B_-crystallin are described in more detail in the Materials and Methods section. As shown in Fig. 5, we see that the resulting values for *D*^L^_s_/*D*_0_ are well described by a power law of the form *D*^L^_s_/*D*_0_ = [(*ϕ*_g_ − *ϕ*)/*ϕ*_g_]^*γ*^, with *ϕ*_g_ = 0.66 and *γ* = 3.7 (see also Fig. S5 in the ESI†). The simulations thus indicate an arrest transition at *ϕ*_g_ ≈ 0.66, and there appears to be no measurable temperature dependence. This is close to the location of the arrest transition for hard ellipsoids with an axial ratio of around 2 found from computer simulations and experiments,^46,47^ and indicates that the weak attraction that drives phase separation and reproduces the structural properties such as the osmotic compressibility has only a weak influence on the long-time dynamic properties such as *D*^L^_s_/*D*_0_ and *η*_r_. Arrest appears to be driven primarily by excluded volume interactions instead.

A similar discrepancy between the model predictions based on computer simulations for weakly attractive spheres or ellipsoids and the experimental observations was previously reported also for the short-time cage diffusion coefficient *D*^S^_c_(q*)/*D*_0_,^18,45^ where the data are also included in Fig. 5. While the simulations predict a concentration dependence that initially follows the behavior of *D*^L^_s_/*D*_0_ and then shows a weaker *ϕ*-dependence at higher concentrations, the experimental data shows a dramatic decrease with increasing *ϕ* already at low volume fractions. Moreover, *D*^S^_c_(q*)/*D*_0_ remains non-zero even in the arrested region, *i.e.* local short-time structural cage relaxations still occur for *ϕ* > *ϕ*_g_. It has previously been suggested that this dramatic slowing down of *D*^S^_c_(q*)/*D*_0_ is the result of patchy attractions leading to the formation of large transient clusters already at low *ϕ*-values.^18^ Computer simulations of patchy particles at comparable values of 
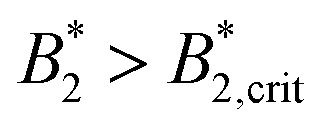
, where 
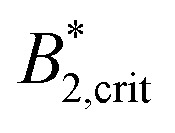
 is the value of 
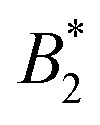
 at the critical temperature, demonstrated that these large clusters can become space-filling already at relatively low *ϕ*-values, resulting in a correspondingly strongly reduced *D*^S^_c_(q*)/*D*_0_. However, while these transient clusters also strongly influence the zero shear viscosity, the strength of the patch–patch attraction results in an estimated bond life time *τ*_b_ ≈ 200 ns that is presumably short compared to the longest stress relaxation time linked to the zero shear viscosity, and thus microscopic dynamical arrest occurs at higher concentrations.^18^

What still remains unclear in such a scenario is why the arrest line appears to be independent of temperature, even though the underlying overall attraction, as indicated by 
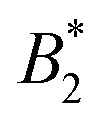
, becomes stronger at lower temperatures. We have studied *γ*_B_-crystallins at relatively high ionic strength, and thus one possible cause could lie in the subtle interplay between different attractive components of the overall interaction potential: on the one hand, a rather weakly attractive isotropic potential (primarily caused by van der Waals attraction) and on the other hand, more strongly attractive patches that could arise either from oppositely charged or hydrophobic patches on the *γ*_B_ surface.^18,48^ If these attractive patches are for example due to hydrophobic interactions, they would lose strength at lower temperatures. At the same time, an overall 
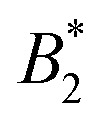
 could still signal stronger overall attraction due to the increasing isotropic contribution. The ratio of the strength of attraction between patches and the isotropic contribution would then also decrease and lead to a change in character for the system. At elevated temperatures, patch attractions would drive cluster formation, which dominates the dynamic properties measured by NSE and microrheology and determines the location of the arrest line. At lower temperatures where patch interactions are smaller, the arrest would follow the typical behavior of particles with isotropic short range attractions. This view is corroborated by previous work, which has demonstrated that at temperatures significantly below the critical temperature *T*_crit_, the arrest line is located in the spinodal region of the phase diagram and shows indeed a significant temperature dependence as expected for an isotropic attractive potential (see Fig. 1).^17^ However, while there are indeed several hydrophobic patches at the protein surfaces, *γ*_B_-crystallin at pH 7.1 also has several charge patches with opposite sign.^49^ Since an increase in temperature also will change the solvent dielectric constant, the Debye screening length and thus the charge distribution and the electrostatic patch–patch interaction, this could also result in an unexpected temperature dependence of the corresponding patch–patch attraction for oppositely charged patches.

## 3. Conclusion

Using tracer particle-based microrheology methods we were able to report the first experimental investigation of the reduced zero-shear viscosity *η*_r_ of *γ*_B_-crystallin solutions over a large volume fraction and temperature range. These measurements have allowed us to precisely locate the arrest line, and compare the concentration and temperature dependence of *η*_r_ with other structural and dynamic properties of *γ*_B_-crystallin. Our results have confirmed the already existing view that this protein can be reasonably well described using a coarse-grained picture of a (patchy) colloid with short range attractions. We observe a measurable critical contribution *η*_crit_ to *η*_r_ in the vicinity of the critical point, in line with the previously characterized divergence of the static and dynamic correlation length measured by light scattering, although the experimentally determined exponent of the corresponding scaling law *η*_r_ ∼ *ε*_*T*_^−(0.12±0.02)^ is considerably larger than the value predicted for classical binary fluids. On the other hand, the overall concentration dependence of *η*_r_ appears to be independent of *T*, in contrast to the large-scale gradient diffusion measured by DLS that is strongly dominated by critical behavior, but similar to the previously reported short time cage diffusion coefficient measured by NSE. The concentration dependence of *η*_r_ is well described by an MCT-like power law over a surprisingly large range of volume fractions, and the magnitude of the exponent in this power law is comparable to what has been found in theoretical studies of colloids with short range attractions. Apparently, while attractive patches cause a dramatic slowing down of cage diffusion, and move the arrest transition to quite low volume fractions, the resulting functional form that describes the concentration dependence of *η*_r_ seems rather insensitive to microstructural details.

There remain some puzzling findings that are not easily understood within this colloid picture. A strongly varying 
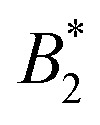
 above the critical temperature, juxtaposed by a temperature independent location of *ϕ*_g_, clearly does not follow the frequently postulated use of an extended law of corresponding states also for non-equilibrium properties such as kinetic arrest.^32^ Obviously dynamic quantities such as short time cage diffusion and viscosity are much more strongly influenced by local details of the interaction potential and the local microstructure than the phase behavior. There is clearly a need for further studies of the link between interaction anisotropy, the formation of transient clusters and their consequences for these dynamic properties. For proteins, this also needs to be linked to the molecular structure and its dependence on the solution properties. We believe that this is not only of fundamental interest for the colloid community, but has consequences for diverse communities such as cell biology or pharmaceutical formulation science, where an ability to predict the existence and location of an arrest transition would be extremely important.

On a more technical level, our study also allows us to draw some conclusions about the potential and limits of these tracer particle-based microrheology techniques. They obviously have significant advantages when compared with classical rheometrical measurements. Traditional rheological measurements of low shear viscosity for low concentration protein solutions are often suffering from artefacts caused by the amphiphilic properties of many proteins, which tend to form a viscoelastic film at liquid–air or liquid–solid interfaces, while measurements at high protein concentrations become prohibitive due to the rather large sample volumes required. This is not the case for tracer particle-based microrheology, which can be performed using DLS and/or MPT. Our study has now provided additional insights into their use in concentrated protein solutions. DLS is for example more robust at low volume fractions and correspondingly low values of *η*_r_, as the resulting high tracer particle mobility only allows for short-time tracking in MPT, leading to decreased statistics and larger error bars. On the other hand, and more importantly, for proteins that exhibit liquid–liquid phase separation the significantly increased protein scattering due to critical fluctuations close to the critical point and the spinodal line causes major problems for DLS-based microrheology, as the extraction of the particle signal becomes significantly less reliable. Naturally, no such effects are visible with MPT, thus when studying systems displaying critical behavior, MPT is strongly recommended over DLS at higher concentrations *c* ≈ *c*_crit_. In this context, using a 3D cross-correlation DLS scheme that suppresses contributions from multiple scattering also is highly advantageous and extends the range of applicability of DLS-based microrheology considerably.

## 4. Materials and Methods

### 4.1. *γ*_B_-Crystallin extraction

The procedure for obtaining and extracting *γ*_B_-crystallin has been described in detail elsewhere.^17,27^ Briefly, the filtered cellular contents of bovine eye-lenses were passed through a size exclusion chromatography column, yielding a mixture of *γ*-crystallins. This mixture was further separated using a fast flow ion exchange column where a salt gradient was used to elute the individual components with *γ*_B_-crystallin being collected. The final sample was added to a 52.4 mM phosphate buffer corresponding to pH 7.1 in D_2_O with the addition of 20 mM dl-dithiothreitol (DTT, Sigma Aldrich D9779), 1 mM ethylenediaminetetraacetic acid disodium salt dehydrate (EDTA, Sigma Aldrich E5134) and 0.02 wt% sodium azide (NaN_3_, Sigma Aldrich S2002). Amicon Ultra centrifugal filter units with a volume of 15 mL (cutoff 3 kDa) or 0.5 mL (cutoff 10 kDa) were used to concentrate the samples. The final protein volume fraction was determined from UV-absorption using an extinction coefficient (0.1%, 1 cm, 280 nm) of 2.18^27^ and converted to a volume fraction using a voluminosity of 0.71 mL g^−1^.^22^

### 4.2. Dynamic light scattering-based microrheology

The first microrheology method follows the motion of tracer particles within the protein sample *via* dynamic light scattering (DLS) and has been thoroughly described earlier.^31,50^ Polystyrene particles with a diameter of 0.3 μm stabilized with covalently-bound 20 kDa poly(ethylene) glycol were then used as tracer particles when mixed with protein samples. DLS measurements at a scattering angle of 90° were performed at 20, 25, 30, and 35 °C. The tracer diffusion coefficient, *D*, was determined from a first order cumulant analysis by fitting a single exponential, when possible, to the field correlation function (*g*_1_), assuming that the scattering intensity was dominated by the tracer contribution and that *g*_1_ directly reflected the tracer motion. In the case when the protein contribution is too significant, the entire correlation function was instead analyzed by simultaneously fitting the combined protein and tracer contribution. Measurements of protein samples without tracers were also performed to verify the result and to make sure that there was no significant long-time protein contribution that may interfere with the tracer fit. The reduced viscosity, *η*_r_, of the sample was extracted using the Stokes–Einstein relation and by normalizing with tracer measurements without protein. A more detailed description of the analysis approach is provided in the ESI.†

### 4.3. Multiple particle tracking-based microrheology

The second microrheology method, multiple particle tracking (MPT), monitors tracer particle motion using videos from confocal laser scanning microscopy (CLSM) and is described in more detail elsewhere.^30^ In short, fluorescently labeled polystyrene particles with a diameter of 1.0 μm (ThermoFisher, F8820) were mixed into samples containing *γ*_B_-crystallin. A 5 μL droplet was transferred to a (cleaned) glass slide and sealed using a SecureSeal spacer (12 mm thickness, Invitrogen) and coverslip. Videos (512 × 256 pixels, 6000 frames, frame rate 77 Hz, 10 μm away from the cover slide) at five different locations in the sample were recorded using a Leica SP5 confocal microscope with a resonant scanner, which is housed in a temperature controlled box, allowing precise control of the temperature. In accordance with DLS measurements, videos were recorded at 20, 25, 30 and 35 °C. The 2D (〈Δ*x*^2^ + Δ*y*^2^〉) mean squared displacement (MSD) of the tracers was calculated using the standard IDL routines^51^ and converted to 3D MSD for generality.^52^ The shown MSDs are time and ensemble-averaged. Rather than extracting the diffusion coefficient from the slope of the MSD – which is quite sensitive to statistics – the Van Hove self-correlation function was calculated. The Van Hove function for one dimension is defined as *P*(Δ*x*,*τ*) = (4π*Dτ*)^−1/2^exp(−〈Δ*x*^2^(*τ*)〉/4*Dτ*).^52,53^ It relates the probability that a tracer moves a distance Δ*x* in a time-step *τ*. For a purely diffusive system (and with sufficient statistics) this probability density function follows a Gaussian distribution.^54^ We tested for which time-step a Gaussian distribution with the highest statistics was obtained, and only used the displacement data at that particular time-step to compute a more robust diffusion coefficient.^53^ The resulting diffusion coefficients in both *x*- and *y*-dimensions were averaged and the viscosity, *η*_0_, was obtained using the Stokes–Einstein relation. To obtain *η*_r_ this viscosity was subsequently normalized using literature values for the viscosity *η*_s_ of D_2_O.^55^

### 4.4. Preparation of solid-like samples

In an effort to reach volume fractions not easily achieved with conventional means we employed the evaporation method described previously.^30^ In these experiments, a controlled amount of time was left between deposition of the sample on the glass slide and sealing of the sticker cell with the coverslip. The amount of solvent that evaporated was determined gravimetrically which allows for determining the increase in protein volume fraction. The samples were then investigated using CLSM as previously established.

### 4.5. Computer simulations

#### 4.5.1. Model description

**Fig. 6:**
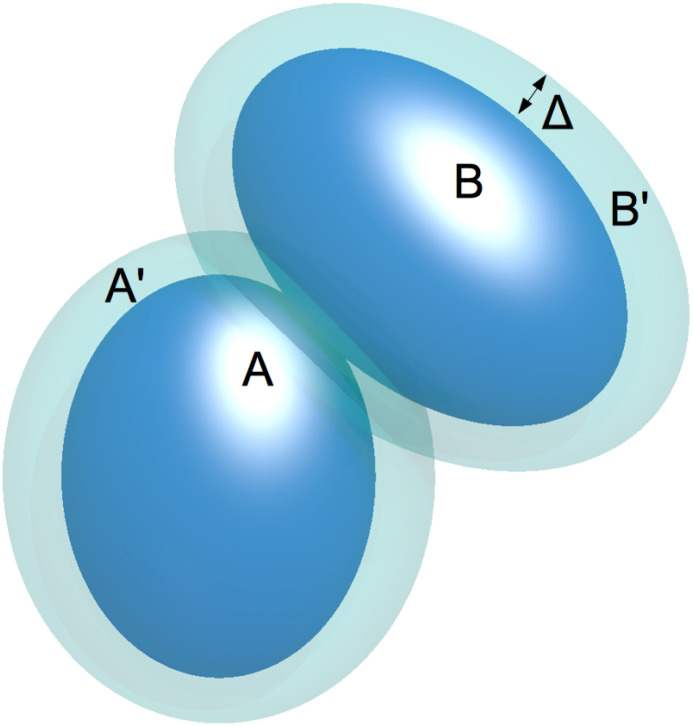
Illustration of the attractive hard ellipsoid (HE) model with its attractive square-well with extension *Δ*.

We carried out Monte Carlo (MC) simulations in the isothermal ensemble (NTV) of a model of the *γ*_B_-crystallin lens protein which has been proposed and used in ref. 17 to study its static properties. The system is constituted of hard ellipsoids (HE) whose semi-axes are *a* = 2.75 nm, *b* = 1.625 nm and *c* = 1.375 nm and which interact through the following square well potential: consider the two HEs A and B, as shown in Fig. 6, and the outer HEs A′ and B′ (transparent ones) whose semi-axes are *a* + *Δ*, *b* + *Δ* and *c* + *Δ* (where *Δ* = 0.6 nm), the A and B interaction energy is 0 if A′ and B′ do not overlap or it is −*ε* if they overlap. For example in Fig. 6 the two HEs A′ and B′ overlap, hence the interaction energy between A and B is −*ε*.

#### 4.5.2. Diffusivity

We studied the reduced temperatures T* = 0.904, 0.94, 0.977 and 1.04, where T* = *k*_B_*T*/*ε*, and for each temperature we performed several NTV-MC simulations covering a range of volume fractions from 0.18 to 0.57. We observed that the choice of the upper volume fraction *ϕ* is motivated by the need to void any coexistence/transition with/to a solid phase. Each simulation is first equilibrated for a sufficient number of steps and then a production run is started during which we collect configurations for later analysis. We checked the equilibration by inspecting the energy of the system. Equilibration runs last up to 3 × 10^6^ MC steps at higher volume fractions.

For all state points we calculated the diffusion coefficient *D* from the mean squared displacement 〈Δ*r*^2^〉, since1
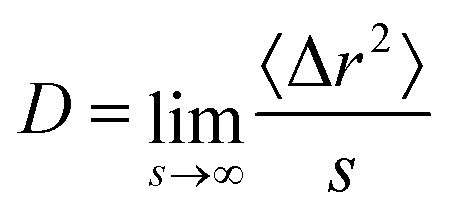
where *s* is the MC step here.

## Data availability

The data supporting this article have been included as part of the ESI.†

## Conflicts of interest

There are no conflicts of interest to declare.

## Supplementary Material

SM-021-D4SM01275E-s001
