# Supplementary material for: Dynamical arrest for globular proteins with patchy attractions
Source: Soft Matter. 2025 Jan 8;21(6):1152–61. doi: 10.1039/d4sm01275e (PMC11740010; doi:10.1039/d4sm01275e)
Supplement: SM-021-D4SM01275E-s001 [file SM-021-D4SM01275E-s001.pdf]

Supplementary Information (SI) for Soft Matter.
This journal is © The Royal Society of Chemistry 2025

Supplementary Information of "Dynamical arrest for globular proteins with patchy attractions"

Maxime J. Bergman,[1] Tommy Garting,[1] Cristiano De Michele,[2] Peter Schurtenberger,[1,3] and Anna Stradner[1,3]

[1] *Division of Physical Chemistry, Department of Chemistry, Lund University, Lund, Sweden*

[2] *Dipartimento di Scienze, Università degli Studi Roma Tre, Via della Vasca Navale 84, Roma, Italy.*

[3] *LINXS - Lund Institute of advanced Neutron and X-ray Science, Scheelevägen 19, SE-223 70 Lund, Sweden.*[a]

[a] Electronic mail: peter.schurtenberger@fkem1.lu.se

DLS ANALYSIS AND DLS AT THE CRITICAL VOLUME FRACTION

The field autocorrelation function g_1 was determined from the intensity autocorrelation function g_2 using the Siegert relation, $g_2(\tau) - 1 = \beta(g_1(\tau))^2$, where β is the intercept. g_1 contains a scattering contribution from both the protein and the tracer, which vary with temperature and concentration. The tracer diffusion coefficient (denoted as D in the main text, but for clarity denoted D_{tracer} here) was then extracted using a single exponential fit to the tracer decay, $g_1(\tau) = Ae^{-q^2 D_{\text{tracer}}\tau}$, when possible. In cases where the protein and tracer decays were too close to conclusively fit only the tracer contribution, a biexponential fit was instead employed including the protein contribution, $g_1(\tau) = Ae^{-q^2 D_{\text{tracer}}\tau} + Be^{-q^2 D_{\text{protein}}\tau}$. In Figure S1 some typical data are shown where especially around ϕ_{crit} fit quality reduces. We note in passing that fitting the protein scattering contribution with a logarithmic decay did not show any significant improvements. The viscosity η of the sample was then obtained via the Stokes-Einstein relation, $D_{\text{tracer}} = \frac{k_{\text{B}}T}{6\pi\eta R_{\text{H}}}$. Here, Boltzmanns constant (k_{B}), temperature (T) and the hydrodynamic radius (R_{H}) of the tracer are all constant, making it straightforward to obtain the relative viscosity η_{r} of the sample by normalizing with tracer measurements without proteins (D_0), so that the calculations for η_{r} simplifies to $\eta_{\text{r}} = D_0/D_{\text{tracer}}$.

Close to ϕ_{crit} the two contributions to $g_1(\tau)$ can not be separated. Therefore, DLS-based microrheology in this volume fraction range was excluded from the final analysis. As can be seen in Figure S2, the viscosities obtained from DLS (squares) in this critical region show deviations with a strong overestimation of the viscosity, which is not echoed in MPT-based microrheology results (circles). This is caused by the overlapping protein and tracer scattering contribution (and concurrent difficulties in separating them in the fitting analysis) as described above and as can be seen in Figure S1. Therefore, we chose to not use these data points for the analysis in the main text but instead show them here for the sake of completeness.

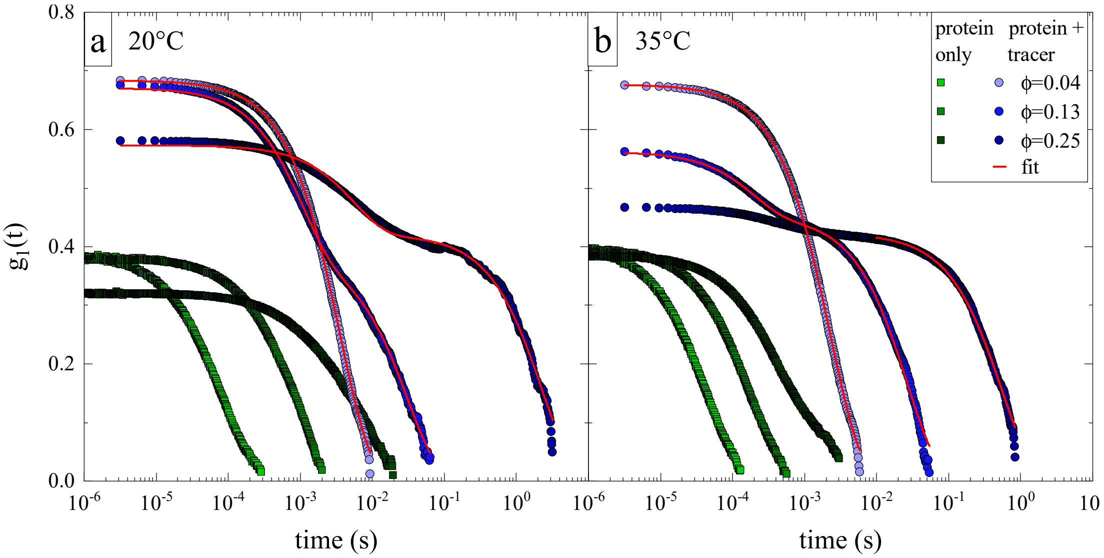

FIG. S1. For completeness' sake, the DLS panels from Figure 1b-c in the main text are repeated here at larger size and including all datapoints for each auto-correlation function.

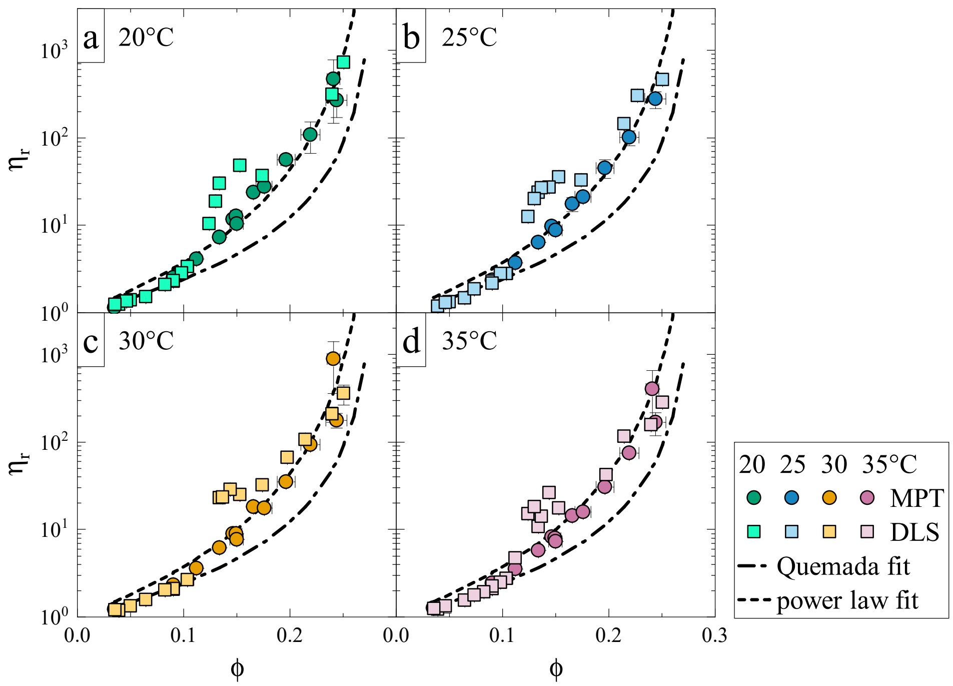

FIG. S2. All reduced viscosity η_r data points obtained with both DLS and MPT-based microrheology. At low volume fraction, both types retrieve similar values. Around ϕ_crit DLS-based data suggests a much higher viscosity due to analysis difficulties. The data from Figure 3 in the main text together with the DLS-results in the intermediate region shown in Figure 1b. The reason for the deviations around the critical region is attributed to poor separation of the two decays in the correlation data.

EVAPORATION CONTROL AND CONFIRMATION OF ARREST

Because the γ_B-crystallin is suspended in buffer, a possible concern is how evaporation increases the salt concentration of the buffer, and whether this affects the arrest transition. Therefore, a protein sample with decreased salt content was created, such that after evaporation, it would have the salt concentration of the original samples. The evaporation procedure was conducted until a volume fraction of $\Phi = 0.32$ was reached. Figure S3, left panel, shows the recorded MSDs of this sample (light blue diamonds) where each trace includes all time-averaged tracking data from one of five videos, recorded at distinctly different positions in the sample. This is compared to the time and location averaged MSD (red squares) of one of the original evaporated samples with nearly the same final volume fraction. The overlap between the two samples provides strong evidence that the change in salt concentration does not have a significant effect on the location of the arrest transition. The outlier MSD was purposely recorded in the small fluid regime that was expelled from the main sample upon sealing.

The right panel in Figure S3 shows the same control sample but remeasured after 24h equilibration (dark blue diamonds). Here, the MSDs are now overlapping with the noise floor (dark line) which is a measure of the instrumental drift. This means that the slight apparent mobility seen in the original measurement was caused by collective motion due to drift inside the sample, most likely generated by the pressure enforced by the cover slip after sealing. After 24h, all such collective motion have halted and the final MSD reflects the true arrest.

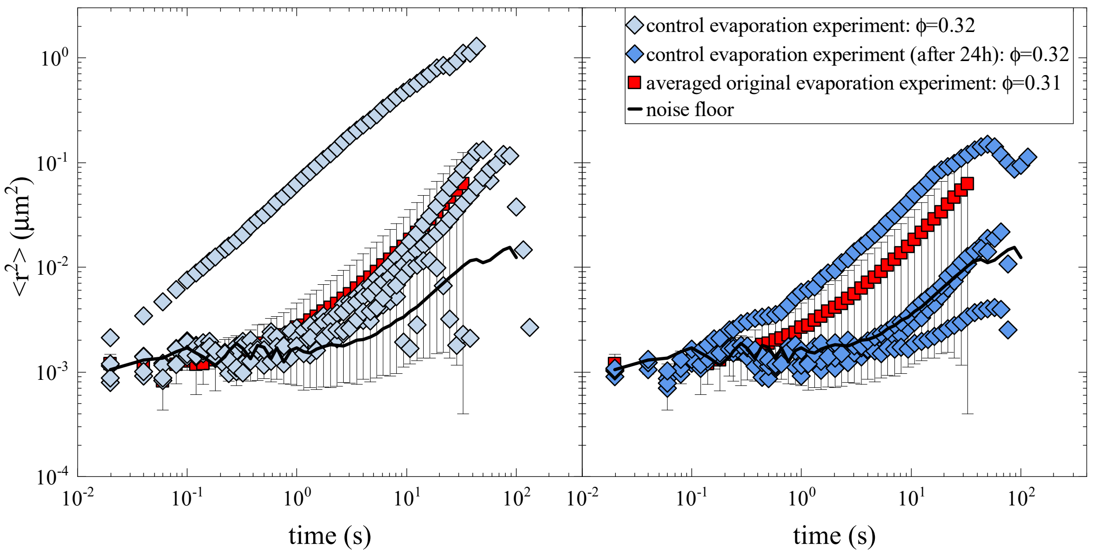

FIG. S3. MSDs from MPT of several different experiments. Light blue diamonds: A control sample created with half the initial buffer strength so that after evaporation it has the original amount. This is to verify that changes in salt concentration due to evaporation do not influence these type of measurements. Dark blue diamonds: The same control sample measured after 24 hour incubation in the sticker cell. This verifies that the apparent diffusive behaviour seen at some locations in the sample is in fact due to macroscopic motion of the sample most likely caused by the pressure of the cover slip. Red squares: The average measurement of the original evaporation experiment showing no significant difference between the two samples. Solid line: noise floor indicating minimum motion detectable in our microscope. The overlap with the dark blue diamonds indicates that the apparent motion at long lag times τ is in fact instrumental drift and that the sample is fully arrested.

ENHANCED VISCOSITY AROUND CRITICAL POINT

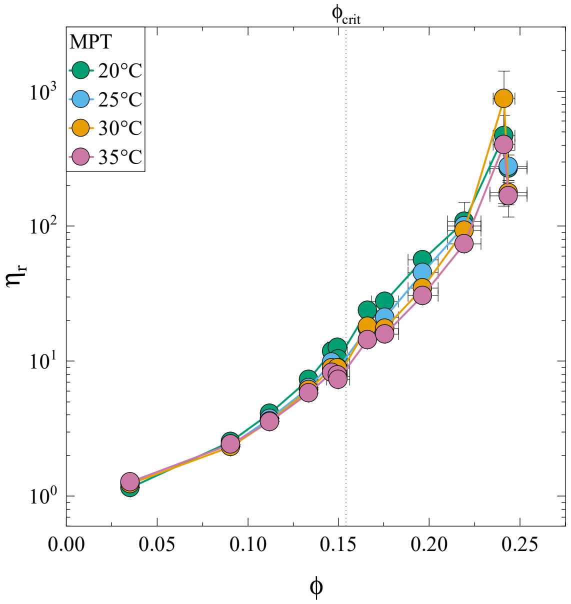

FIG. S4. Reduced viscosity η_r obtained with MPT-based microrheology at 20-35°C (as indicated by legend). The data shows a weak temperature effect around $\phi_{crit} = 0.154$. Upon approaching $T_{crit} \approx 19$°C, samples become more viscous due to critical contributions. Surprisingly, as we show in the main text, samples display a larger viscosity than expected based on universal scaling laws, suggesting additional contributors to the viscosity.

COMPUTER SIMULATIONS

For all state points we calculated the diffusion coefficient D from the mean squared displacement $\langle \Delta r^2 \rangle$, since

$$D = \lim_{s\to\infty} \frac{\langle \Delta r^2 \rangle}{s} \tag{1}$$

where s is the MC step here. The diffusion coefficients as a function of ϕ for all the temperatures studied are shown in Fig. 5 in the main text and they exhibit a weak dependence on temperature so that all curves almost collapse one on top of each other. This result suggests that dynamic at moderate and high volume fractions for this model is mostly controlled by steric hindrance of HEs.

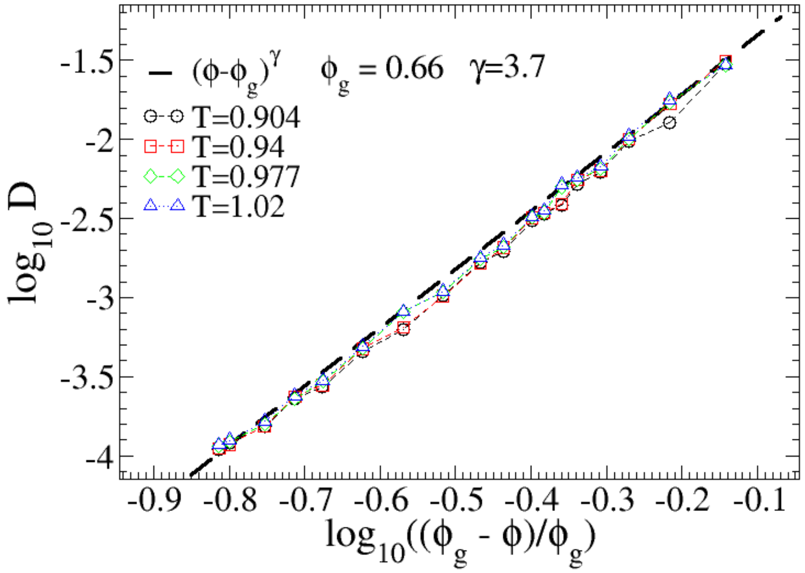

FIG. S5. Logarithm of the self diffusion coefficient D from computer simulations of the HE model as a function of the logarithm of the reduced volume fraction $(\phi_g - \phi)/\phi_g$, where $\phi_g = 0.66$, for the reduced temperatures $T^* = 0.904$, 0.94, 0.977 and 1.04, where $T^* = k_B T/\epsilon$. Also shown as a dashed line is the power law relationship $D = D_0[(\phi_g - \phi)/\phi_g]^\gamma$ with $\gamma = 3.7$.

We also find that the volume fraction dependence of D obtained in these simulations, when plotted in a log-log plot, all fall on a straight line, indicating a power law dependence on concentration of the form $D = D_0[(\phi_g - \phi)/\phi_g]^\gamma$ with $\gamma = 3.7$ (as shown in Fig. S5), which suggests that a possible glass transition may occur at concentrations much larger than

those observed experimentally for γ_B-crystallin, although the critical exponent is in line with experimental findings.
